# Doctors vs. Algorithms: Physicians, too, struggle to learn from evidence that contradicts AI suggestions

**DOI:** 10.1371/journal.pdig.0001490

**Published:** 2026-07-09

**Authors:** Aranzazu Vinas, Fernando Blanco, Helena Matute

**Affiliations:** 1 Department of Economics and Management, University of the Basque Country, Spain; 2 Department of Social Psychology, University of Granada, Spain; 3 Mind, Brain and Behavior Research Center (CIMCyC), Granada, Spain; 4 Department of Psychology, University of Deusto, Spain; National Tsing Hua University, TAIWAN

## Abstract

Despite their widespread adoption, Artificial Intelligence-based Patient Classification Systems sometimes rely on incorrect, outdated, or incomplete data, which can lead to inaccurate outputs. Nevertheless, health professionals are expected to override these errors, at least when they have access to critical information. To test this, we conducted two experiments in which professional physicians interacted with an Artificial Intelligence system that incorrectly classified fictitious patients as either highly or lowly sensitive to a treatment. The physicians administered the treatment to a series of fictitious patients and received feedback that was useful for learning that the patient classification was incorrect and that all patients were equally sensitive to the treatment. We ran two experiments: in Experiment 1, the medicine showed medium effectiveness for both types of patients, while in Experiment 2, the treatment was completely ineffective for both types of patients. The results showed that, in the two experiments, physicians generally trusted the AI-based patient classification and struggled to learn from the evidence. Furthermore, in Experiment 2, they failed to realize that the treatment was ineffective. Our findings have important implications for healthcare professionals and patients, underscoring the need to critically evaluate Patient Classification Systems.

## Author summary

Artificial Intelligence (AI) is now widely used in various fields, including clinical practice, where AI systems assist in classifying patients for diagnosis and treatment. However, these AI-based patient classification systems can sometimes use incorrect, outdated, or incomplete data, leading to errors. In such cases, health professionals are expected to catch and correct these errors. Earlier studies found that the general population often do not sufficiently detect and fix AI’s mistakes. We wanted to see if professional physicians would act differently so that they would notice and correct these errors. To find out, we conducted two experiments in which participants had to decide whether to administer a fictitious treatment to patients who were incorrectly classified by an AI as being more or less sensitive. In the first experiment, the treatment worked moderately (and equally) well for both groups. In the second, the treatment did not work at all for either group. In both experiments, physicians trusted the AI’s classifications and did not use the available information to override the erroneous classification. What is more, in the second experiment, they did not even realize that the treatment was completely ineffective.

## Introduction

As in many other fields, the use of Artificial Intelligence (AI) is becoming widespread in different areas of clinical practice [1]. Since its introduction, the potential of AI to revolutionize healthcare practice appears to be huge. Thus, AI is expected to greatly contribute to the improvement of diagnostic accuracy [2,3,4], the facilitation of treatments [5], and the prediction of drug response [6,7,8]. Among the many applications of AI for diagnosis and treatment delivery, one of its most used functionalities in recent years has been patient classification [1,7,9]. Patient classification refers to the process of categorizing patients based on their care needs. In this sense, AI systems use certain patient characteristics or symptoms to generate patient categories and assist in classifying individuals accordingly. Then, health professionals can use this classification to make better decisions, whether recommending a treatment or requesting additional diagnostic tests.

However, despite the growth in their use, AI Patient Classification Systems -like other classification systems- can be fraught with errors, so the accuracy of the recommendations and predictions might sometimes, as a result, be compromised [7,8]. In these situations, we should expect human supervisors to correct mistakes in the AI’s output. In fact, the European Commission (2024) [10] has developed the AI Act, which establishes that in high-risk domains, such as healthcare, AI systems cannot operate autonomously, and human actors are expected to oversee AI outputs, validating or rejecting them. Indeed, some of the previous research provides evidence that, when humans interact with AIs, a synergistic relationship emerges so that the results are better than those achieved by either humans or AIs alone (see for example [Vaccaro et al., 2024 and Takita et al., 2025, and Cabitza et al., 2023 and Zöller et al., 2025 [11,12,13,14] for specific results in the area of health). Nevertheless, those results are usually based on studies conducted with AIs showing low error rates. However, it is not strange that AIs exhibit poorer accuracy levels in certain applications and through the different stages of the clinical process [15]. For example, when the models are trained with homogenous data sets while the actual populations are diverse [16]. The error rates also tend to be relatively high in intuitive and ambiguous decision-making tasks [17], and in those that require a high level of creativity [18], or empathy [19]. In this regard, previous research suggests that people tend to not sufficiently correct the AI’s mistakes, leading to biases in judgments and decisions (see for example Agudo et al., 2024; Jacobs et al., 2021 and Vicente & Matute, 2023 [20,21,22]).

In line with the previous argument, recent research conducted by Vinas et al. (2025) [23] demonstrated the danger of human over-reliance on Patient Classification Systems. The authors conducted two experiments in which the participants (who were anonymous Internet users, rather than professional doctors) had to decide whether to administer a treatment to a series of fictitious patients classified as highly or lowly sensitive to the treatment. Importantly, this classification was incorrect, as both groups of patients healed with the same probability. Immediately after administering the treatment to each patient, the participants were informed whether the patient had healed. Thus, participants had the opportunity to learn that the classification system was not accurate by just looking at the outcomes of their decisions. After visiting all the patients, the participants judged to which extent they believed the treatment was effective for each type of patient. The authors found that the misclassification of patients not only influenced the amount of treatment that the participants administered to each type of patient, but more importantly, the participants were unable to learn from the evidence that consistently showed that patients were cured to the same extent, independently of the classification. As a result, participants incorrectly judged the treatment to be more effective in patients classified as highly sensitive.

The reasons why participants showed this pattern of behavior may comprise several related processes. First, automation bias [24], which drives participants to the default response of believing the AI indication without question. Second, their decisions on whether to use the medicine on some of the patients can be affected by confirmation bias [25]. Thus, they would use the treatment specifically in those situations in which it is expected to work better, hence biasing the information they observe. Finally, the combination of frequently treated patients and frequent recoveries can lead to causal illusions, that is, an overestimation of the treatment effectiveness [26].

The previous result may not seem surprising considering that the participants in Vinas et al.’s (2025) [23] study were not medical experts. Thus, an important question remains unanswered: what would happen if the participants were medical professionals? Would they also be swayed by the misclassification, or would they use the correct information provided in the task to override the incorrect AI classification?

Some of the available evidence suggests that experts think and make decisions differently from novices. Their learning processes tend to be more active and systematic [27], and they can access relevant knowledge more quickly [28,29]. As a consequence, experts appear to be more effective at filtering out irrelevant information [28,29], identifying appropriate problem-solving strategies [29], making predictions [30,28], and reducing errors [31,28]. Nevertheless, numerous studies have shown that experts’ judgments and decisions are not always better than those of novices. For example, clinicians have been found to occasionally fail into cognitive biases similar to those of the general population, such as automation bias [32], overconfidence and confirmation bias [33,34,35]. This might suggest, for instance, that experts’ causal learning processes, which are often based on heuristics, may not differ much from those of novices’, so they will probably fall into the same type of cognitive biases that are common in the general population when judging causal relationships (e.g., Matute et al., 2015 [36]). That is, professionals, just as everyone else, could judge that causality exists in cases where both the potential cause and the outcome are frequent, overriding the available evidence that points to the contrary, and without taking into account all the necessary information [26].

The previous argument motivates our current research, in which we ask whether medical doctors would be able to learn from experience about the effectiveness of a fictitious treatment when they are given the opportunity to use it on a series of fictitious patients. To address this question, we built upon the procedure used by Vinas et al. (2025)[23], to which we introduced two modifications. First, we conducted our experiments with professional physicians rather than anonymous Internet volunteers. Second, due to the increasing use of AI in medicine, and because we wanted to explore its implications, the patient classification in our experiments was explicitly described as AI-based.

As in Vinas et al. (2025) [23], the two experiments reported herein differed in the real effectiveness of the fictitious treatment: in Experiment 1, it was equally mildly effective across the different patient types, whereas in Experiment 2, it was equally ineffective for all patients (thus aiming to model a pseudomedicine). We predicted that, in both experiments, participants would administer the treatment less often to the fictitious patients classified as lowly sensitive to the treatment, consistently with the AI classification. The information on the recovery rates of each patient type could then be used to challenge the erroneous classification system. At the same time, we predicted that participants would judge the treatment as less effective for patients classified as lowly sensitive than for those classified as highly sensitive, as participants would see less recoveries associated to the first patients. That is, we expected that the incorrect recommendation made by the AI would not be overridden by actual evidence, hindering the ability to learn the causal relationship between treatment and patient recovery, as it had happened with the general population sample in the original study [23].

Finally, since AI elicits conflicting opinions regarding its applicability and effectiveness [3], and considering that prior attitudes are known to influence behavior and judgments regardless of expertise [37], we expected that participants with more positive general attitude toward AIs, and those who perceived the AI used in the experiments as more reliable, would exhibit a stronger classification effect (i.e., a greater difference between the effectiveness judgments for patients classified as highly sensitive and for those classified as lowly sensitive).

## Experiment 1

### Method

#### Ethic statement.

The Ethical Review Board of the University of Deusto approved the procedure of Experiments 1 and 2. We performed them in accordance with the approved guidelines. All participants signed the informed consent. We did not collect any personal or identifiable data.

#### Participants.

We recruited 120 anonymous participants via the Prolific survey platform. The sample size was decided based on economic reasons. A sensitivity analysis conducted before data collection indicated that with 120 participants, we could detect a small effect size, *f* = 0.129, with 80% power in the 2x2 interaction. We limited the sample to people speaking fluent English, who had not participated in previous experiments conducted by our research group and, importantly, who worked as doctors in the healthcare sector. Because it was the first time we used the Prolific professional filter, we additionally introduced two questions as a double control measure (“Do you work in the healthcare sector?” and “Do you work as a doctor?”). We predefined that we would discard participants who either declared not working in the healthcare sector or not being medical doctors. However, none of the participants in our sample answered negatively these questions and therefore none was excluded for this reason. Additionally, we asked participants what their professional experience (in years) was, and their specialty. Since the latter questions were collected on an open textbox, we later recoded the specialty following the WHO classification [38]. Finally, we also pre-registered that we would eliminate participants who incorrectly answered any of two control questions, one at the end of the instructions (thus ensuring the participants’ attention), and one in the AI attitudes questionnaire. Six participants incorrectly answered the control question in the instructions, and another nine participants incorrectly answered the control question in the questionnaire. Therefore, the final sample after exclusions was *N* = 105 (distribution by gender: 58 women and 47 men; age: *M* = 38.6, *SD* = 13.3). The mean professional experience in years was *M* = 13.1, *SD* = 11.3, and the most common specialties were General Medicine (*n* = 28, i.e., 27% of the participants), and Pediatrics (*n* = 9, i.e., 9% of the participants). The study pre-registration is available at https://aspredicted.org/dp9z-3dgm.pdf.

#### Procedure.

The experimental task was based on the one used in the study by Vinas et al. (2025) [23] described in the Introduction section. At the beginning of the experiment, the participants read the instructions. We explained that they had to imagine that they were doctors treating a rare disease with a treatment that was still under development, so its effectiveness had not yet been proven. We used a fictious treatment and disease because we did not want to bias the participants by using real stimuli with which they might have previous experience. We also informed the participants that they would see a series of patients classified by an AI agent as either highly or lowly sensitive to the treatment. That is, according to the classification, some patients (highly sensitive) were expected to improve with higher probability than others (lowly sensitive) by using the treatment. We gave no additional information about the AI classification system, since trustworthy and authority effects might depend on how an AI system is framed (the instructions are available at https://osf.io/6nkrm).

After reading the instructions, the participants proceeded to the learning phase, in which they were presented with a sequence of 60 patients in random order: 30 classified as highly sensitive and 30 classified as lowly sensitive. Each trial portrayed an individual patient. The information we gave to participants was that each individual patient was suffering from Lyndsay syndrome and the label assigned to each one by the AI (either highly or lowly sensitive to the treatment). First, the participants had to choose whether the treatment should be administered to that patient. Immediately afterward, the screen informed whether the patient healed or not. Unbeknownst to the participants and independent of the patient category, 70% of the patients healed when they received the drug, and only 20% of them healed when they did not receive it. This means that the drug was an effective treatment, but more importantly, it was identically effective for both categories of patients. That is, the categorization of patients given by the AI was incorrect. No further information about patients or context (e.g., comorbidities, disease trajectory, patient history, etc.) was provided, in order to ensure that the only information that was available was the combination of AI labels, treatment given, and outcome observed. This ensures that the decisions were made by the participants under uncertainty, and that external information and prior knowledge were not affecting them.

We collected two dependent variables. The first was P(C), or the probability with which participants administered the drug on each category of patients. This is calculated as the number of doses given to each of the two patient categories divided by the total number of patients in the category (i.e., 30). Therefore, P(C) could take values between 0 and 1. Once the participants had visited all 60 patients, we also collected their treatment effectiveness judgment for each patient category. We presented the two questions on the same screen and randomized their position (top/ down) for each participant. The judgments were given on a scale from 0 (completely ineffective) to 50 (moderately effective) to 100 (completely effective). In this particular experiment, the correct answer was 50, because the actual effectiveness of the drug can be computed as the difference between the probability of healing when the drug is used (70%) minus the probability of healing when the drug is not used (20%).

After collecting the effectiveness judgments, we asked participants to rate the extent to which they perceived the AI used in the experiment to be reliable. Specifically, they completed the sentence: “I believe that the classification of patients performed by the AI has been...” with one of 5 possible answers: (1) Very unreliable, (2) Unreliable, (3) Neutral, (4) Somewhat reliable, and (5) Very Reliable.

Finally, for measuring the attitudes toward AI, we used the General Attitudes toward AI Scale, GAAIS, [39], which contains 20 items (12 for the positive attitude factor and eight for the negative attitude factor) plus one item controlling attention. All items were rated from 1 (strongly disagree) to 5 (strongly agree).

## Results and discussion

### Influence of perceived reliability and attitudes toward AI on P(C) and effectiveness judgments

Although the perceived reliability and attitudes toward AI were not our key variables, we preferred to analyse their impact first, in order to see whether we needed to take them into account when performing our more critical analyses on how the patient classification system influenced the P(C) and the effectiveness judgments. The perceived reliability of the AI was *M* = 3.65 (*SD* = 1.05) out of a maximum score of five, which means it was overall above the midpoint. Additionally, the two subscales of the GAAIS (on a scale from 0 to 5) yielded *M* = 3.7 (*SD* = 0.696) for the positive attitude, and *M* = 2.9 (*SD* = 0.627) for the negative attitude. Both subscales were negatively correlated, *r* = -0.42, *p* < .001 (i.e., those with stronger positive attitude tended to show weaker negative attitude, and vice versa). Furthermore, the perceived reliability question correlated significantly with the GAAIS scale of positive attitude toward AI, *r* = 0.374, *p* < .001, although not with the negative attitude scale, *r* = -0.012, *p* = .905.

To test whether both perceived reliability of AI and patient classification jointly influenced P(C), we conducted a repeated measures ANOVA with patient classification (highly sensitive, lowly sensitive) as a within-subject factor and AI reliability as a covariate. We found a significant effect of patient classification on P(C), *F*(1, 97) = 5.639, *p* = .02, *η²*_*p*_ = .055. However, neither reliability, *F*(1, 97) = 2.28, *p* = .135, *η²*_*p*_ = .023, nor the interaction between reliability and patient classification, *F*(1, 97) = 0.039, *p* = .843, *η²*_*p*_ = .0, showed significant effects on P(C).

We ran a similar analysis to test whether both patient classification and AI attitude were jointly influencing P(C), that is, an ANOVA with patient classification (highly sensitive, lowly sensitive) as factor and two covariates: GAAIS positive attitude, and GAAIS negative attitude. We found a significant effect of patient classification on P(C), *F*(1, 102) = 8.74, *p* = .004, *η²*_*p*_ = .079. However, neither positive attitude to AI, *F*(1,102) = 0.129, *p* = .72, *η²*_*p*_ = .001, nor negative attitude to AI, *F*(1,102) = 1.179, *p* = .28, *η²*_*p*_ = .011, nor the interaction between these and patient classification, *F*(1,102) = 3.28, *p* = .073, *η²*_*p*_ = .031, and *F*(1,102) = 2.15, *p* = .146, *η²*_*p*_ = .021, showed significant effects on P(C).

We expected that participants with a more positive general attitude toward AIs, and those who perceived the AI as more reliable, would exhibit a stronger classification effect. Contrary to this hypothesis, neither the perceived reliability nor the attitudes toward AI influenced the probability with which physicians administered the treatment. This null result can be attributed to different factors that will be later discussed. Nevertheless, we continued to analyze the data without considering these two variables.

### Influence of the Patient category on P(C) and effectiveness judgments

**Fig 1:**
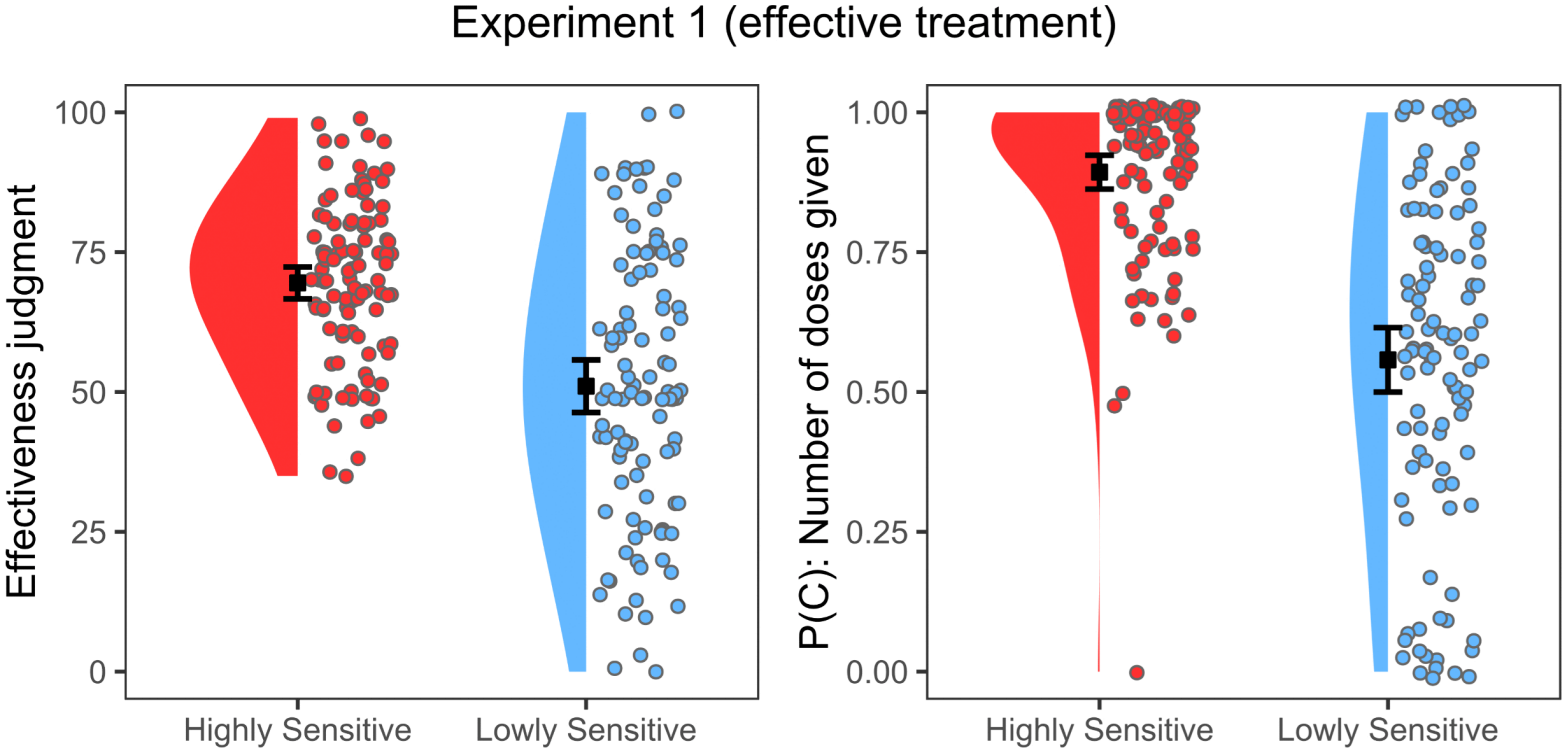
Mean P(C) and Mean Judgment of Effectiveness as a function of Patient Classification in Experiment 1. Error bars depict 95% CIs for the mean. The P(C) shows the probability with which the treatment is administered.

Fig 1 shows the critical data in this experiment. The P(C) for highly sensitive patients was *M* = 0.893 (*SD* = 0.156) while it was *M* = 0.557 (*SD* = 0.298) for lowly sensitive patients. We pre-registered a repeated measures ANOVA for this experiment with patient type as within-subjects factor. As the t-test is equivalent to the pre-registered analysis but is simpler to report, we decided to report it instead. The conclusions are exactly the same. The Paired samples t-test showed that the patient classification significantly influenced the P(C), *t*(104) = 9.35, *p* < .001, *d* = .912, that is, participants administered the treatment more often to those patients classified as highly sensitive. In addition, and regarding the effectiveness judgment, the mean values were *M* = 69.5 (*SD* = 14.7) for highly sensitive patients and *M* = 51 (*SD* = 24.3) for lowly sensitive patients. As in the case of P(C), a Paired samples t-test showed that the Patient Classification System significantly influenced the effectiveness judgments, *t*(104) = 7.19, *p* < .001, *d* = .702, so par*t*icipants judged that the treatment was more effective in those patients classified as highly sensitive.

Furthermore, a correlational analysis showed that the P(C) for highly sensitive patients did not significantly correlate with the causal judgment for these patients, *r* = 0.147, *p* = .134, whereas the P(C) for lowly sensitive patients positively and significantly correlated with the causal judgment of these patients, *r* = 0.4, *p* < .001. That is, whereas in the highly sensitive patients the efficacy judgments did not depend on the number of doses administered, in the case of lowly sensitive patients the efficacy judgments increased with the number of doses. Although this experimental design does not allow to prove mediation or causation, these results are consistent with those found by Vinas et al. (2025) [23], suggesting that the effectiveness judgments differ between the patient types as a result of administering different amounts of drug doses to patients classified as highly sensitive and lowly sensitive to the treatment.

## Experiment 2

In Experiment 1, we examined the effect of an incorrect AI Patient Classification System on the administration and effectiveness judgment of an effective drug, by a sample of professional physicians. First, we expected that those participants who considered that the AI was highly reliable would trust more the classification provided by the AI system, and as a result, they would administer more treatment doses to the patients who were incorrectly classified as highly sensitive. Second, we expected that participants showing a strong positive attitude toward AI would also administer more treatment doses to the patients classified as highly sensitive. As a result, we expected that the judgment of drug effectiveness would be higher for patients classified as highly sensitive. Contrary to our expectations, we did not find either that perceived reliability and attitude toward AI influenced the quantity of treatment administrated or the effectiveness judgments. In fact, we found that the incorrect AI patient classification influenced the majority of participants’ decisions, independently of their attitudes and their perceptions about the reliability of the AI. As a consequence, in general, most physicians in our sample administered more treatment doses to those patients incorrectly classified as highly sensitive, and consistently, their judgments of effectiveness were also higher for these patients.

In Experiment 2, we tested whether the incorrect patient classification provided by an AI agent would also influence physicians when they were confronted with pseudomedicine (i.e., a treatment presented as potentially useful but not proven effective. Note that although the term “pseudomedicine” is commonly used to refer to a concept with numerous clinical, legal, and ethical implications, in this study we focus exclusively on the aspect of empirical scientific evidence. Accordingly, our laboratory model of pseudomedicine (which is used solely to study participants’ perceptions of effectiveness) consists of a fictitious treatment presented as potentially effective but lacking empirical evidence of efficacy and showing no correlation with patients’ recovery. Given that in our procedure the participants have access to crucial information (i.e., the co-occurrences between treatment and recovery) that can inform the perception of the treatment effectiveness, overriding the incorrect suggestion made by the AI system, it is possible that they could learn that the treatment is actually ineffective for all patients. However, previous research has shown that it is also possible for people to develop illusions of causality [36], thereby believing that ineffective treatments work, particularly when the rate of spontaneous recovery of the disease is high [26]. So, in this case we tested whether it was also possible that the participants followed the recommendation made by the AI system, just as it happened in the previous study where the treatment was indeed effective. If this was the case, we would expect participants to administer more doses of the ineffective treatment to patients classified as highly sensitive and to develop a higher illusion for those patients. Indeed, Vinas et al. (2025) [23] found that this was the case with a general sample of participants. Here, we expected to replicate the same result with professional physicians.

## Method

### Participants

We aimed to recruit 120 participants via the survey platform Prolific. In this case, we also limited the sample to people speaking fluent English who had not participated in previous experiments conducted by our research group (including Experiment 1) and who worked as medical doctors in the healthcare sector. In this experiment, we also introduced the same two questions to ensure that the professional filters were correct (“Do you work in the healthcare sector?” and “Do you work as a doctor?”), and the same attention control question as in Experiment 1 placed at the end of the instructions. We planned the exclusion criteria as in Experiment 1 (participants declaring not working in the healthcare sector or not being medical doctors), but none was excluded for these reasons. Due to technical errors, we finally recruited 121 participants (one more than planned). In addition, three participants incorrectly answered the control question in the instructions, thus leaving the final sample as *N* = 118 (distribution by gender: 58 women and 47 men; age: *M* = 36.6, *SD* = 12.6). The mean professional experience in years was *M* = 10.6, *SD* = 9.4, and the most common specialties were General Medicine (*n* = 34, i.e., 28% of the participants), and Internal Medicine (*n* = 13, i.e., 11% of the participants). The study pre-registration is available at https://aspredicted.org/mbww-mfcc.pdf.

### Procedure

The experimental task was similar to that of Experiment 1, with the following two changes: (1) First, the most important modification was that, in this case, 70% of patients were programmed to recover when they received the treatment, but the same proportion of patients (70%) recovered when they did not receive it. In other words, the treatment was not an effective drug but a pseudomedicine, because it was unable to increase the probability of healing when it was used compared to when it was not used. A high percentage of recovery was used because previous research has shown that the illusion of causality is favoured in these cases [26]. Consequently, in this experiment, the correct judgment of effectiveness was zero. (2) Because we had found that reliability and attitudes toward AI did not influence either treatment administration or effectiveness judgments in Experiment 1, we eliminated these variables in this second study.

### Results and discussion

**Fig 2:**
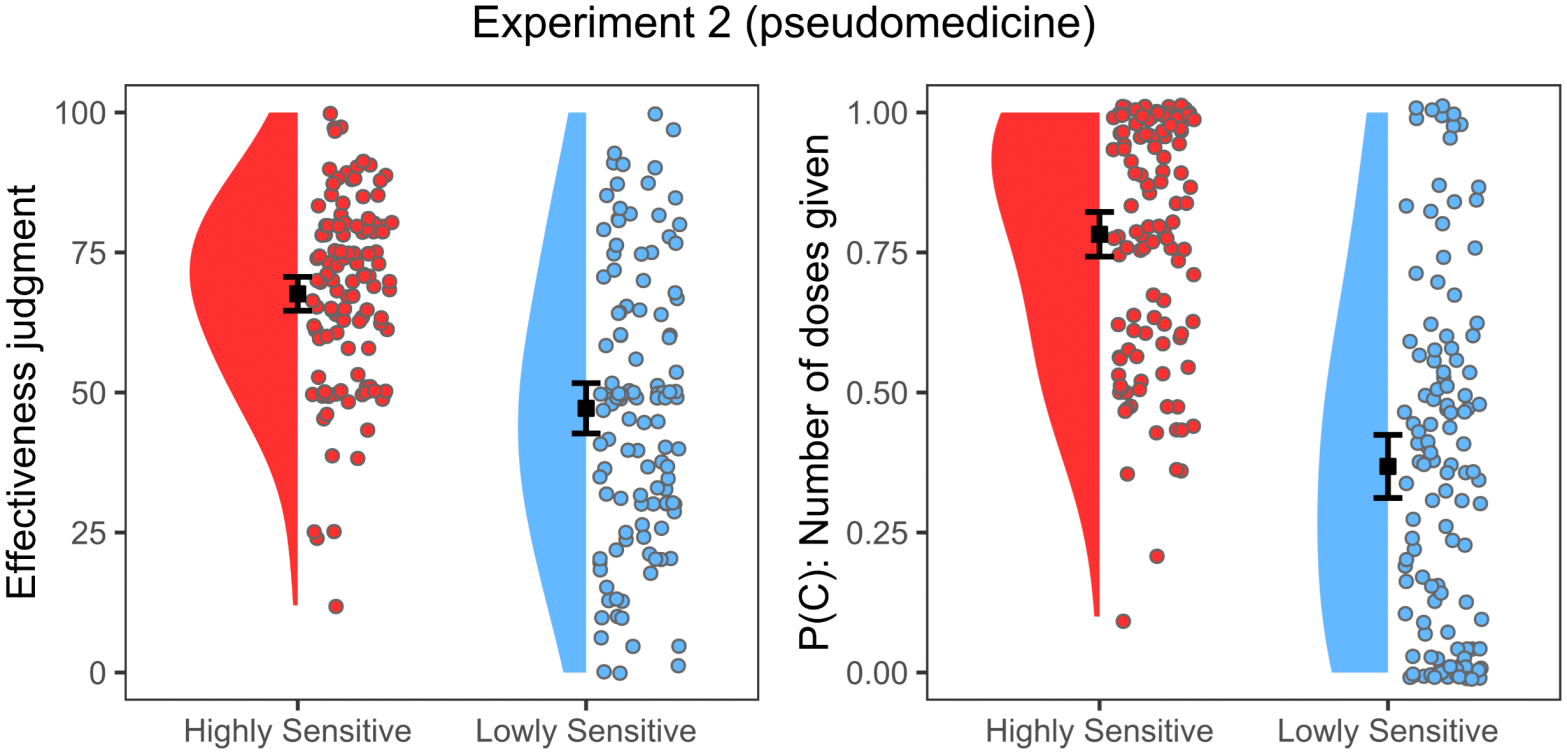
Mean P(C) and Mean Judgment of Effectiveness as a function of Patient Classification in Experiment 2. Error bars depict 95% CIs for the mean. The P(C) shows the probability with which the treatment is administered.

Fig 2 depicts the P(C) and effectiveness judgments for patients classified as highly and lowly sensitive. The P(C) for highly sensitive patients was *M* = 0.783 (*SD* = 0.218) whereas for lowly sensitive patients it was *M* = 0.368 (*SD* = 0.309). A Paired samples t-test showed that the AI classification significantly influenced P(C), *t*(117) = 10.29, *p* < .001, *d* = 0.947, that is, participants administered the pseudomedicine more often to patients classified as highly sensitive.

Fig 2 also shows the effectiveness judgments for highly sensitive patients, *M* = 67.6 (*SD* = 16.6), and for lowly sensitive patients, *M* = 47.2 (*SD* = 24.7). In both cases, the judgments were significantly higher than zero: for patients classified as highly sensitive, *t*(117) = 44.3, *p* < .001, *d* = 4.08, and for patients classified as lowly sensitive, *t*(117) = 20.8, *p* < .001, *d* = .1.91. This indica*t*es that participants generally overestimated the effectiveness of a treatment that was, in fact, completely ineffective (i.e., they showed an illusion of causality). They did so even when they had the opportunity to learn from the evidence from each patient. Additionally, we also ran a Paired samples t-test which showed that the AI-guided classification significantly influenced the judgments, *t*(117) = 7.44, *p* < .001, *d* = .685, such tha*t* participants gave higher effectiveness judgments for patients classified as highly sensitive than for patients classified as lowly sensitive.

Finally, a correlational analysis showed that, in this case, the P(C) directly correlated with the effectiveness judgment for both types of patients, *r* = 0.234, *p* = .011 for patients classified as highly sensitive, and *r* = 0.568, *p* < .001 for patients classified as lowly sensitive. These results suggest that, when faced with a pseudomedicine, a higher (or lower) effectiveness judgment might be the result of administering a higher (or lower) quantity of treatment, on average, for both types of patients, as shown also with a regular population sample by Vinas et al. (2025) [23].

## General discussion

In this research, our goal was to investigate whether physicians would rely on their preconceptions about patients, as induced by an erroneous AI-Patient Classification System, or whether they would learn from the empirical evidence showing that this classification was incorrect and that the rate of healing was equal independently of the type of patient. Vinas et al. (2025) [23] had already shown that the general population relies on incorrect classifications and, thus, has problems when learning causal relations from their experience, as a result of not considering all the available evidence when judging causality. The current research shows that the same happened with medical doctors faced with an erroneous AI recommendation. As a result of the incorrect AI recommendation, participants administered the treatment more often to patients classified by the AI as highly sensitive, and their effectiveness judgments for the treatment were also higher for these patients. Moreover, the classification outweighed the evidence presented during the session in the form of patient recoveries, which means that most participants did not use this information to learn and override the incorrect classification provided by the AI. This was found when the treatment was equally effective for all participants (Experiment 1), but, perhaps more interestingly, the same result was found when the treatment was completely ineffective for all patients (Experiment 2). Thus, participants in Experiment 2 made two mistakes: believing that the classification was right and believing that an ineffective treatment worked, and they did so despite the necessary information being available for them. These results replicate those observed by Vinas et al. (2025) [23] with the general population. More specifically, most people (and now physicians as well) (1) did not use the evidence available from recoveries that contradicted the AI classification, and (2) developed an illusion of causality with respect to the efficacy of the treatment in those patients that the AI had classified as more sensitive (i.e., this occurred even when the evidence from patients showed that the treatment was totally ineffective in Experiment 2). This we shows that physicians had the same tendency to rely on the AI recommendations as other people, even when the available evidence showed that these recommendations were incorrect.The previous results provide evidence of how given classifications can shape behavior and judgments. As Bowker and Star (2000) [40] state, classifying is a fundamental human practice: all cultures, throughout history, have produced classification systems and, even though people do not always realize it, classification systems are influential, often without awareness. The influence of classifications can also override other sources of knowledge, such as empirical evidence in the form of observed patient recovery rates.

Contrary to one of our predictions, we failed to find significant effects of the explicit attitudes toward AI in general and the perceived reliability of the (fictitious) AI mentioned in the study. However, our measures were based on explicit self-reports. It could also be the case that the participants’ explicit and implicit attitudes were misaligned. Since we did not measure implicit attitudes, we cannot be sure that they are not affecting the results. In fact, previous research states that people can hold different attitudes at the same time, one implicit and unrecognized and the other one explicit and conscious, so implicit attitudes, being automatically activated, can often guide behavior by default unless they are overridden by controlled processes [41]. This has also been found specifically in the field of technology (see for example [42,43,44]. Thus, it could be interesting for future research to measure implicit attitudes toward technology, to test: (1) whether these can drive both behavior and judgments, (2) whether implicit and explicit attitudes toward technology can be misaligned, and (3) whether the influence of the patient classification can indeed outweigh that of implicit attitudes.

In our two experiments, we provided participants with the specific piece of information they needed to realize that the AI classification was wrong, namely the patient recovery rates. Participants seemed to neglect this information. Thus, apparently, physicians did not learn from available evidence that the AI classification was incorrect, and that the treatment was equally effective for all patients in Experiment 1. Moreover, we consider the result of Experiment 2 even more dramatic as participants did not realize either that the classification was incorrect nor that the treatment was completely ineffective. It is important to note that the consequences for patients are different depending on the actual effectiveness of treatment. That is, in Experiment 1, when the treatment is effective, the problem with trusting more the AI classifications than the available evidence is that a good, useful treatment is not given to a certain group of patients who would benefit from it, thus creating a potential discrimination toward this group. In Experiment 2, the problem is that, because the classification is believed and not questioned by the evidence, an ineffective treatment is given to patients and an illusion of causality emerges (i.e., most participants end up believing that the treatment works). This would have important consequences in real life, not only in economic and time terms, but above all, in terms of health risks due to the opportunity cost of possibly replacing a valid treatment with a pseudomedicine [45].

These studies have several limitations that must be discussed. First, a potential limitation is the reliance on self-reported professional status. The primary disadvantage of self-reports is limited credibility, which may affect the accuracy of responses. However, self-reported data regarding profession, specialty, and years of experience are objective and not very susceptible to self-deception or memory lapses, which are common sources of error in self-reports [46]. Nevertheless, as previously mentioned, to enhance data quality, we asked these questions in our experiment, in addition to the questionnaire provided by Prolific.

Another potential limitation concerns the ecological validity and the generalizability of laboratory experiments. These experiments allow us to control for extraneous variables (e.g., previous knowledge about the disease, contextual information…), thus helping us to make the causal claim that it is the misclassification of patients what influences both the decision to use the treatment and the effectiveness judgments, while other factors such as for example different levels of previous knowledge about the disease are not affecting the results. However, it is possible that the materials used are overly artificial and simplistic to grant strong generalization to real-life decisions made by physicians as a part of their job. Therefore, as the results may be task-dependent, we advise caution when applying these conclusions to real-world decisions until more ecological research is conducted in the health context. Next, we outline several aspects that need to be considered in further research to improve ecological validity.

To begin with, generalisation to real-world clinical settings in which AI is used should acknowledge the diversity of situations, contexts, and experiences that we find in real life. For example, the present research examines medical qualification alone, rather than specific expertise in evaluating empirical evidence or AI systems under uncertainty. When extending it to real healthcare contexts, it would be interesting to analyse whether a specific specialty and/or training in clinical research or experimental medicine might moderate susceptibility to incorrect AI classification effects. Specifically, most physicians (from specialities such as General Medicine) are trained to work within well-stablished, evidence-based classification systems, guidelines, and protocols, and are encouraged not to challenge them unless there is a strong reason. This makes sense in most contexts because protocols and systems are designed and tested rigorously, and individual doctors access a more limited set of information to make decisions, therefore they are instructed to prioritise consistency, safety, and protocol adherence. By contrast, physicians from some specialties with strong clinical research training and more experience with experimental methods might be more likely to engage in hypothesis-testing and collect evidence from patients´ recovery to draw conclusions, perhaps challenging the proposed AI classification.

A second aspect that is simplified in our experiments is the type and amount of information available to participants. It is important to bear in mind that in practice, clinicians typically access multiple sources of patient information such as patient history, comorbidities, and disease trajectory, rather than relying solely on the AI classifications and the clinical outcomes resulting from the application (or not) of the treatment to each patient. Clinical decisions frequently involve more than two possible actions, rather than simple binary choices (administer/ not administer the treatment). Moreover, we did not provide a detailed description of the AI classification process or clarify whether the system had undergone prior testing, that is, whether it had been validated or was experimental. This lack of specificity may have influenced the results. User trust and decisions may vary depending on whether the AI is presented as validated or experimental, or as highly or minimally sophisticated [47]. Because we did not provide this information about whether the AI system was still experimental or had been validated, it seems sensible to assume that participants probably believed that it had already been validated and was free of errors. As a result, they may have followed its recommendations, as is often the case in real clinical contexts when a new AI system is introduced. In addition, several aspects of the design of the AI system are also known to play a role in decision making. For example, the number of classifications provided and the degree of explainability can also influence user responses [48]. The workflow design is also important: in our experiments, the AI system delivered its recommendation prior to user decision-making, which reflects the most common real-world application. However, alternative designs exist, such as requiring users to provide an initial opinion before receiving the AI recommendation (see [20], for a discussion of simultaneous versus sequential designs). Extending this research to real healthcare settings would enable comparisons of how specific features of different real AI systems impact human-AI interactions and how these interactions are moderated by human expertise and experience. Quite possible, if the AI-classification system had been labelled as experimental, or under construction, participants would have weighted more seriously the evidence that the outcomes from the individual patients was suggesting. However, and even though the unreliable status of the AI classification system in our experiment was not explicitly stated, we believe it is still quite worrisome that the participants did not incorporate the evidence they received about the recoveries of the patients and did not realize that the treatment was equally effective (or ineffective) for all patients, and therefore that the AI classification system was erroneous.

Despite these limitations to the generalization, the study models clinician – AI interaction under maximal epistemic uncertainty (i.e., lack of contextual information that could affect the results in real life). We judge our results to be relevant mainly for two reasons. First, we have proven that in uncertain situations, a Patient Classification System can override the available evidence about causality and treatment efficacy. When judging treatment effectiveness, physicians based their judgments mostly on the AI Patient Classification System, without completely integrating the evidence on real healing rates, a relevant piece of information that was available to them. The results can be then interpreted in two ways: either our participants showed the same cognitive bias found in non-experts, or they strategically relied on the protocols under epistemic uncertainty. Certainly, in real-life situations, this behavior could reflect adaptive calibration of confidence, as most clinicians are trained to work within established classification systems, protocols, and guidelines, and to treat them as authoritative unless there is a strong and relevant reason to challenge them (as previously mentioned). In the current experiments, participants were confronted with a fictitious disease and a fictitious treatment. Since neither the disease nor the treatment corresponded to the participants’ prior clinical knowledge, clinicians could not rely on the usual cognitive scaffolding that supports critical evaluation in real clinical practice. This raises the possibility that the participants’ behavior reflected a contextually appropriate deference to an external decision support system in the absence of specific knowledge about the disease, rather than a failure of learning from evidence. That is, it could be argued that the findings cannot completely distinguish between cognitive bias and adaptive behavior in participants’ following the AI recommendations. However, the finding that an erroneous patient classification system can override immediately available and relevant evidence (i.e., recovery rates) under conditions of epistemic uncertainty (i.e., lack of context, previous knowledge on the disease, and so on) can still have negative consequences for patients in real clinical contexts. Whereas it might be considered as adaptive behavior to follow the recommendation of an AI system in some contexts, even if it is erroneous, the evidence on recovery rates that was available should have warned participants that something was wrong with the system, and at least their efficacy judgments should have been more evidence-based. That is, the AI classification in the current experiments affected not only the participants’ behavior, but also their subjective judgments on the efficacy of the treatment, which is something they should have inferred correctly if they had taken into account the available evidence on recovery rates.

Second, as previous research has shown, people in general are more willing to accept advice when they trust the advisor [49]. At the same time, not only people in general, but also health professionals in particular, seem to have the tendency to over rely on automated decision-making systems (see [24] and [32] for an example). Precisely for that reason, we believe that the fact that the classification is based on an erroneous AI can be particularly problematic, as the presence of an AI recommendation could increase the problem of the influence of misclassification on professionals’ decisions.
